# The efficacy and safety of pertuzumab plus trastuzumab and docetaxel as a first-line therapy in Japanese patients with inoperable or recurrent HER2-positive breast cancer: the COMACHI study

**DOI:** 10.1007/s10549-020-05921-x

**Published:** 2020-09-13

**Authors:** Masato Takahashi, Shoichiro Ohtani, Shigenori E. Nagai, Seiki Takashima, Miki Yamaguchi, Michiko Tsuneizumi, Yoshifumi Komoike, Tomofumi Osako, Yoshinori Ito, Masahiko Ikeda, Kazushige Ishida, Takahiro Nakayama, Tsutomu Takashima, Takashi Asakawa, Sho Matsumoto, Daisuke Shimizu, Norikazu Masuda

**Affiliations:** 1 Department of Breast Surgery, National Hospital Organization Hokkaido Cancer Center, Sapporo, Japan; 2 Breast Surgery, Hiroshima City Hiroshima Citizens Hospital, Hiroshima, Japan; 3 Breast Oncology, Saitama Cancer Center, Saitama, Japan; 4 Breast Oncology, National Hospital Organization Shikoku Cancer Center, Matsuyama, Japan; 5 Breast Surgery, JCHO Kurume General Hospital, 21 Kushihara-machi Kurume, Fukuoka, Japan; 6 Breast Surgery, Shizuoka General Hospital, Shizuoka, Japan; 7 Surgery, Kindai University Hospital, Osakasayama, Japan; 8 Breast Center, Kumamoto Shinto General Hospital, Kumamoto, Japan; 9 Breast Medical Oncology, Cancer Institute Hospital of JFCR, Tokyo, Japan; 10 Breast and Thyroid Surgery, Fukuyama City Hospital, Hiroshima, Japan; 11 Surgery, Iwate Medical University, 2-1-1, Idaidori, Yahaba-cho, Shiwa-gun, Iwate Prefecture, 028-3695 Japan; 12 Breast and Endocrine Surgery, Osaka International Cancer Institute, 3-1-69 Otemae, Chuo-ku, Osaka, 541-8567 Japan; 13 Breast and Endocrine Surgery, Osaka City University Graduate School of Medicine, 1-4-3 Asahimachi Abeno-ku, Osaka, 5458585 Japan; 14 Clinical Information and Intelligence Department, Chugai Pharmaceutical Co., Ltd, 1-1 Nihonbashi-Muromachi 2-Chome, Chuo-ku, Tokyo, 103-8324 Japan; 15 Clinical Study Management Department, Chugai Pharmaceutical Co., Ltd, 1-1 Nihonbashi-Muromachi 2-Chome, Chuo-ku, Tokyo, 103-8324 Japan; 16 Clinical Science and Strategy Department, Chugai Pharmaceutical Co., Ltd, 1-1 Nihonbashi-Muromachi 2-Chome, Chuo-ku, Tokyo, 103-8324 Japan; 17 Surgery, Breast Oncology, National Hospital Organization Osaka National Hospital, Osaka, Japan

**Keywords:** Pertuzumab, Trastuzumab, Docetaxel, HER2-positive inoperable/recurrent/advanced/metastatic breast cancer, Prospective clinical trial, Japanese patients

## Abstract

**Purpose:**

In the CLEOPATRA study of patients with human epidermal growth factor receptor 2 (HER2)-positive recurrent or metastatic breast cancer, the Japanese patient subgroup did not demonstrate the improved progression-free survival (PFS) of pertuzumab plus trastuzumab and docetaxel vs. placebo that was seen in the overall population. Therefore, COMACHI was conducted to confirm the efficacy and safety of this treatment regimen in this patient subgroup.

**Methods:**

This was a phase IV study of pertuzumab plus trastuzumab and docetaxel in Japanese patients with histologically/cytologically confirmed inoperable or recurrent HER2-positive breast cancer. All patients received pertuzumab, trastuzumab, and docetaxel intravenously every 3 weeks until disease progression/unacceptable toxicity. The primary endpoint was investigator-assessed PFS. Secondary endpoints were overall survival (OS), investigator-assessed objective response rate, and duration of response (DoR). Safety was also assessed.

**Results:**

At final analysis, median investigator-assessed PFS was 22.8 months (95% CI 16.9–37.5). From first dose, OS rate at 1 year was 97.7%; and at 2 and 3 years were 88.5% and 79.1%, respectively. Of the 118 patients with measurable disease at baseline, response rate was 83.9% (95% CI 77.3–90.5) and median investigator-assessed DoR was 26.3 months (95% CI 17.1–not evaluable). Treatment was well tolerated, with no new safety signals detected.

**Conclusions:**

Our results suggest similar efficacy and safety for pertuzumab plus trastuzumab and docetaxel in Japanese patients compared with the overall population of CLEOPATRA, providing further support for this combination therapy as standard of care for Japanese patients with inoperable or recurrent HER2-positive breast cancer.

**Electronic supplementary material:**

The online version of this article (10.1007/s10549-020-05921-x) contains supplementary material, which is available to authorized users.

## Introduction

Breast cancer is the second most common type of cancer and was the third most common cause of cancer-related mortality in 2018, accounting for 2,088,849 (11.6%) of new cancer diagnoses and 626,679 (6.6%) cancer-related deaths worldwide [1]. In Japan, there were 87,050 new cases of breast cancer reported in 2015 and 14,285 related deaths in 2017 [2, 3]. Overexpression or amplification of human epidermal growth factor receptor 2 (HER2) occurs in approximately one-fifth of all breast cancers and has been associated with a poor prognosis historically [4, 5]. Introduction of anti-HER2 agents for the treatment of patients with HER2-positive metastatic breast cancer has improved outcomes (median overall survival [OS] and 5-year survival rates) in these patients [6].

Pertuzumab plus trastuzumab and a taxane is now the recommended standard of care for the first-line treatment of patients with HER2-positive recurrent or metastatic breast cancer in both the National Comprehensive Cancer Network and the Japanese Breast Cancer Society Clinical Practice Guidelines [7, 8]. Dual HER2 blockade with pertuzumab plus trastuzumab and docetaxel as a first-line treatment for HER2-positive recurrent or metastatic breast cancer demonstrated superiority in progression-free survival (PFS), as assessed by an independent review committee (IRC), and in OS in the global phase III, randomized, placebo-controlled Clinical Evaluation of Pertuzumab and Trastuzumab (CLEOPATRA) study (ClinicalTrials.gov number, NCT00567190) [9, 10]. Median IRC-assessed PFS (the primary endpoint) in the intention-to-treat population was improved by 6.1 months in the pertuzumab arm compared with the placebo arm (18.5 months vs. 12.4 months; stratified hazard ratio (HR) 0.62; 95% confidence interval (CI) 0.51–0.75; stratified log-rank *P* < 0.001) [9]. At the time of the PFS primary analysis, OS did not meet the stopping criteria for significance [9]. However, after an additional year of follow-up, superiority in OS was demonstrated [10]; median OS was not reached in the pertuzumab arm but was 37.6 months in the placebo arm (HR 0.66; 95% CI 0.52–0.84; *P* < 0.001) [10]. In the final analyses at a median follow-up of 99 months, median PFS and median OS in the pertuzumab arm were improved by 6.3 months (HR 0.69; 95% CI 0.59–0.81) and 16.3 months (HR 0.69; 95% CI 0.58–0.82), respectively [11].

However, in the primary analysis of PFS in CLEOPATRA, consistency of efficacy was not confirmed in Japanese patients [12]. Of the 26 patients included in the pertuzumab arm and the 27 patients included in the placebo arm, 14 (53.8%) and 11 (40.7%) patients experienced a PFS event, respectively, and median PFS was 12.5 months in the pertuzumab arm vs. 18.7 months in the placebo arm (stratified HR 1.63; 95% CI 0.70–3.78) [12]. In light of this, Japan’s Pharmaceuticals and Medical Devices Agency has since recommended confirmation of the efficacy of pertuzumab in Japanese patients [12]. Therefore, the COMACHI study (clinicaltrials.jp identifier, JapicCTI-132321) was conducted to confirm the efficacy and safety of pertuzumab plus trastuzumab and docetaxel as a first-line therapy for Japanese patients with inoperable or recurrent HER2-positive breast cancer.

## Methods

### Study design

This was a phase IV, multicenter, single-arm study of pertuzumab plus trastuzumab and docetaxel in Japanese patients. Although the study was not a randomized, comparative study, key design components such as eligibility criteria and definition of endpoints were similar to those seen in the pertuzumab arm in the CLEOPATRA study [9].

Eligible patients were females aged ≥ 20 years who had histologically or cytologically confirmed inoperable or recurrent HER2-positive breast cancer (immunohistochemistry 3 + or in situ hybridization *HER2*: chromosome enumeration probe 17 ratio ≥ 2.0) and were candidates for chemotherapy, or had unresectable locally recurrent or new stage IV HER2-positive breast cancer. Additional inclusion criteria were the presence of measurable or unmeasurable lesions per the Response Evaluation Criteria In Solid Tumors (RECIST) version 1.1, a baseline left ventricular ejection fraction (LVEF) of ≥ 50%, an Eastern Cooperative Oncology Group (ECOG) performance status of 0 or 1, and the use of appropriate contraception during and for ≥ 7 months after the last dose of study treatment. Key exclusion criteria were the identification of metastases to the central nervous system, a history of LVEF decline to < 50% during or after neoadjuvant/adjuvant trastuzumab therapy, a history of anticancer treatment for inoperable or recurrent breast cancer (epidermal growth factor receptor/anti-HER2 drug/vaccine, chemotherapy, two or more prior hormonal regimens), treatment history of approved or investigational tyrosine kinase/HER family inhibitors for breast cancer at any time (except for trastuzumab as neoadjuvant/adjuvant therapy), having prior systemic therapy for breast cancer as neoadjuvant/adjuvant therapy and a disease-free interval of < 12 months from the date of last taxane administration in neoadjuvant/adjuvant therapy until diagnosis of recurrence, and prior exposure to a cumulative dose of doxorubicin or liposomal doxorubicin that exceeded 360 mg per square meter of body-surface area or its equivalent.

All patients included in the study received a loading dose of pertuzumab (840 mg) and trastuzumab (8 mg/kg), followed by a maintenance dose (420 mg and 6 mg/kg, respectively) intravenously every 3 weeks until disease progression or unacceptable toxicity. Docetaxel (75 mg/m^2^) was also administered to all patients intravenously every 3 weeks. The recommended number of cycles for docetaxel was at least six; until Cycle 6, docetaxel was discontinued only if disease progression or uncontrollable adverse events occurred, and continuation of docetaxel beyond Cycle 6 was at the discretion of the investigator.

The study was performed in accordance with the Declaration of Helsinki, the International Conference on Harmonisation E6 Good Clinical Practice (ICH-GCP-E6) guidelines, and local laws and regulations. Approval for the study protocol, any protocol amendments, and all material provided to the patients were obtained from the relevant institutional review board or ethics committee at each site. All patients provided written informed consent.

### Endpoints

The primary endpoint was investigator-assessed PFS. Secondary endpoints were OS, investigator-assessed objective response rate (ORR), and duration of response (DoR). Safety and tolerability were also assessed. Disease control rate (the percentage of patients who achieved complete response [CR], partial response [PR], or stable disease) and investigator-assessed time to response were exploratory endpoints.

Investigator-assessed PFS was defined as the time from first dose to the first documented radiographic evidence of progressive disease according to RECIST v1.1, or death from any cause within 18 weeks after the last independent assessment of tumors. OS was defined as the time from first dose to death from any cause, and investigator-assessed ORR as the rate of PR or CR occurring after first dose and confirmed ≥ 28 days later per RECIST v1.1.

### Assessments

Tumor assessments were performed every 9 weeks for the first 2 years, and then every 18 weeks thereafter, in accordance with RECIST v1.1, until the time of disease progression or death. Treatment decisions were made by the investigator on the basis of their assessment of disease progression.

LVEF assessments were performed at baseline, every 9 weeks during the treatment period, at the time of treatment discontinuation, and then every 18 weeks or less thereafter. Final LVEF assessments were performed 28–42 days after final administration.

Adverse events were monitored continuously throughout the study and were graded per the National Cancer Institute’s Common Terminology Criteria for Adverse Events, version 4.03. Patients with adverse events leading to treatment discontinuation were followed for as long as possible, until resolution of the event or stabilization of the patient’s condition.

### Statistical methods

The target sample size for the study was 130 patients to ensure 80% statistical power at a two-sided significance level of 0.05 to reject the null hypothesis that the median PFS in the COMACHI study was less than 12.4 months, which was the observed median IRC-assessed PFS in the placebo arm of the primary analysis in the CLEOPATRA study [9]. Efficacy analyses were planned for patients in the intention-to-treat population, which included all patients who were enrolled in the study, and safety analyses for all patients who received ≥ 1 dose of a study drug. We also conducted an exploratory analysis of median PFS and 95% CI by demographic subgroups, and an exploratory, post hoc analysis of the efficacy of pertuzumab plus trastuzumab as a maintenance therapy in patients who intentionally discontinued docetaxel beyond Cycle 6.

All patients received treatment and were analyzed for both efficacy and safety. The Kaplan–Meier method was used to estimate the medians and the distribution of PFS, OS, and DoR. CI for medians was calculated using the Brookmeyer–Crowley method of log–log transformation. CIs for response rates were calculated using the Clopper–Pearson method. Safety analyses were descriptive.

## Results

### Patients

A total of 132 patients were enrolled in the study across 29 sites in Japan between November 2013 and September 2015. Primary analysis was performed to satisfy the protocol-defined trigger for primary analysis; the date of clinical cutoff for the primary analysis was June 7, 2018, with a median follow-up of 24.9 months. Final analysis was performed because it was concluded that there would not be a significant gain of information with a further follow-up, and the sponsor consequently closed the study. The clinical cutoff date for the final analysis was April 25, 2019, with a median follow-up of 46.9 months.

Patient demographics and baseline characteristics are shown in Table 1. The median age of patients was 56.5 years (range 34–81 years). The majority of patients had an ECOG performance status of 0 and a HER2 status of immunohistochemistry 3 + . Seventy (53.0%) patients were estrogen receptor-positive; 71 patients (53.8%) had stage IV disease and 81 patients (61.4%) had visceral metastasis at baseline. Approximately one-fifth of patients had received treatment with trastuzumab prior to study enrollment.

**Table 1:** Patient demographics and baseline characteristics in the intention-to-treat population

Characteristic	Patients (*N* = 132)
Female, *n* (%)	132 (100)
Median age, years (range)	56.5 (34–81)
ECOG performance status, *n* (%)	
0	115 (87.1)
1	17 (12.9)
Stage of disease at initial diagnosis, *n* (%)	
Stage I	8 (6.1)
Stage II	31 (23.5)
Stage IIIA	7 (5.3)
Stage IIIB	7 (5.3)
Stage IIIC	8 (6.1)
Stage IV	71 (53.8)
Site of disease, *n* (%)	
Non-visceral	51 (38.6)
Visceral	81 (61.4)
Hormone-receptor status, *n* (%)	
ER- and/or PgR-positive	72 (54.5)
ER- and PgR-negative	60 (45.5)
HER2 status by IHC/ISH, *n* (%)	
IHC 2 + and ISH-positive	17 (12.9)
IHC 3 + and ISH-positive IHC 3 + and ISH unknown	4 (3.0) 107 (81.1)
IHC unknown and ISH-positive	4 (3.0)
Recurrence	
De novo metastatic disease	83 (62.9)
Recurrent metastatic disease^a^	49 (37.1)
Disease-free interval (month)^b,c^	
*n*	49
Mean (SD)	55.7 (41.7)
Median	44.3
Min–max	0.6–179.4
Prior therapies, *n* (%)	
No	94 (71.2)
Yes	38 (28.8)
Anthracycline	25 (18.9)
Taxanes	22 (16.7)
Trastuzumab	30 (22.7)

*ECOG* Eastern Cooperative Oncology Group, *ER* estrogen receptor, *HER2* human epidermal growth factor receptor 2, *IHC* immunohistochemistry, *ISH* in situ hybridization, *PgR* progesterone receptor

^a^Patients with non-recurrence were classified as having "de novo metastatic disease”

^b^Disease-free interval is the time from completion of systemic treatment (chemotherapy) and/or surgery to the diagnosis of metastatic/recurrence disease

^c^Patients with a disease-free interval of < 1 month were not assumed to have “de novo metastatic disease”

### Progression-free survival (primary endpoint)

**Fig. 1:**
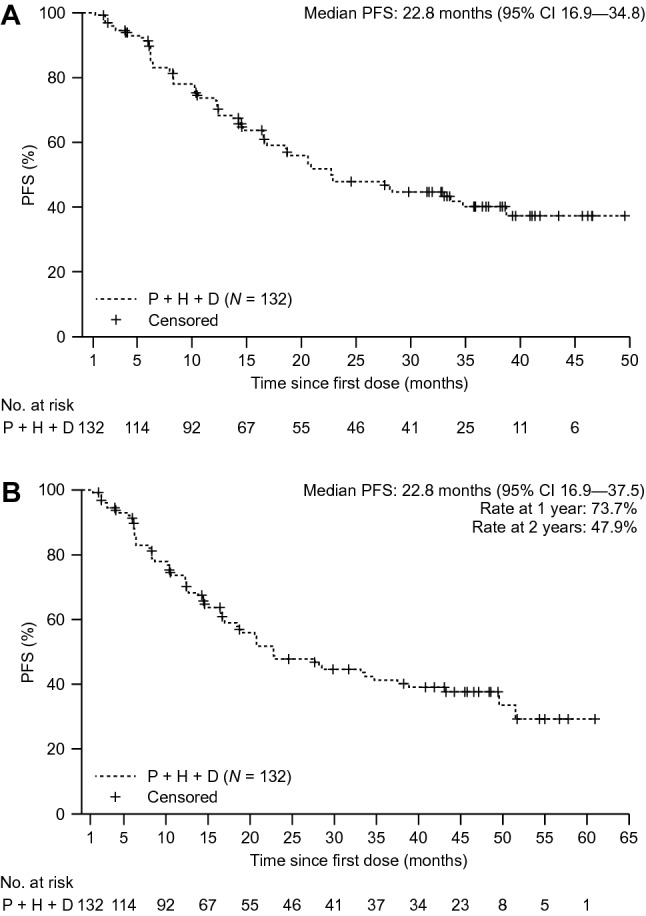
Investigator-assessed PFS at the **a** primary analysis at the clinical cutoff date of June 7, 2018 and **b** final analysis at the clinical cutoff date of April 25, 2019

At the primary and the final analyses, median investigator-assessed PFS was 22.8 months (primary analysis: 95% CI 16.9–34.8; final analysis: 95% CI 16.9–37.5) (Fig. 1). The lower limit of the 95% CI for median investigator-assessed PFS was above the preset threshold of 12.4 months; therefore, a median PFS of at least 12.4 months was demonstrated. From first dose of the study drug, PFS rates at 1 year and 2 years were 73.7% and 47.9%, respectively (Fig. 1b).

### Key secondary endpoints

**Fig. 2:**
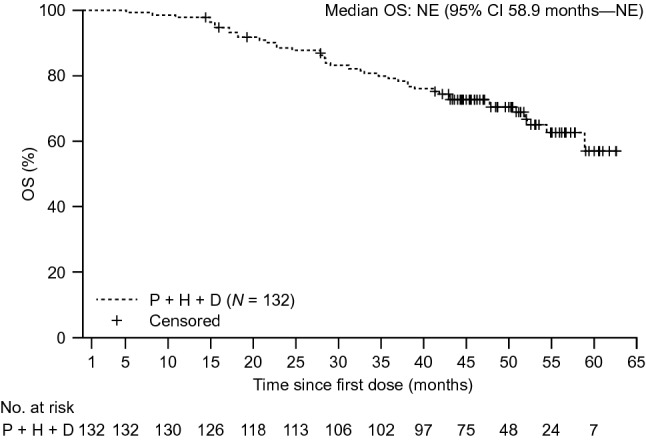
OS at the final analysis (clinical cutoff date: April 25, 2019)

Median OS was not reached at the final analysis (Fig. 2) due to the small number of events (*n* = 42 patients [31.8%]). From first dose, OS rate at 1 year was 97.7% and at 2 and 3 years was 88.5% and 79.1%, respectively (Fig. 2).

Of the 118 patients with measurable disease at baseline, investigator-assessed response rate was 83.9% (95% CI 77.3–90.5), with a best overall response of CR in 11 patients (9.3%) and PR in 88 patients (74.6%) (Table 2). Median investigator-assessed time to response was 2.1 months (95% CI 2.0–2.1) and median investigator-assessed DoR was 26.3 months (95% CI 17.1–not evaluable [NE]). A further 16 patients (13.6%) had stable disease, giving a disease control rate of 97.5% (95% CI 94.6–100.0); three patients (2.5%) had progressive disease.

**Table 2:** Best overall response in patients with measurable disease at baseline (*n* = 118)

Best overall response	Patients, *n* (% [95% CI]) (*n* = 118)
Objective response	99 (83.9 [77.3–90.5)
Complete response	11 (9.3 [4.08–14.6])
Partial response	88 (74.6 [66.7–82.4])
Stable disease	16 (13.6 [7.38–19.74])
Progressive disease	3 (2.5 [0.00–5.38])

*CI* confidence interval

#### Exploratory analysis of efficacy

At the final analysis, estimated median PFS in each of the demographic subgroups was similar to that of the overall Japanese population (Table 3), with the exception of hormone-receptor status (positive and negative hormone-receptor status: median PFS = 18.9 months [*n* = 41; 95% CI 14.7–33.1] vs. 33.7 months [*n* = 29; 95% CI 20.6–NE]; Online Resource 1) and site of disease (non-visceral and visceral: median PFS = 37.5 months [*n* = 19; 95% CI 20.7– NE] vs. 18.4 months [*n* = 51; 95% CI 13.8–33.7]; Online Resource 2). Kaplan–Meier plots of median PFS for other demographic subgroups are shown in the supplementary materials (Online Resource 3–7). Compared with patients who continued docetaxel treatment beyond Cycle 6, discontinuation of docetaxel at Cycle 6 did not impact PFS irrespective of whether discontinuation was due to adverse event/lack of efficacy or not (Online Resource 8 and 9).

**Table 3:** Subgroup analyses of progression-free survival

	*n*	Number of events, *n* (%)	Median PFS, months (95% CI)	HR (95% CI)
Overall population	132	70 (53.0)	22.8 (16.9–37.5)	–
Prior (neo)adjuvant therapy^a^				
Yes	38	18 (47.4)	27.7 (12.4–NE)	0.91 (0.53–1.56)
No	94	52 (55.3)	20.8 (16.9–38.8)	–
Age				
< 65	96	53 (55.2)	22.8 (16.5–37.5)	1.29 (0.75–2.23)
≥ 65	36	17 (47.2)	33.1 (18.7–NE)	–
Site of disease				
Non-visceral	51	19 (37.3)	37.5 (20.7–NE)	0.62 (0.37–1.06)
Visceral	81	51 (63.0)	18.4 (13.8–33.7)	–
HER2 status IHC/ISH				
IHC ≤ 2 + and ISH-positive	21	13 (61.9)	16.5 (10.4–34.8)	–
IHC 3 +	111	57 (51.4)	22.9 (18.4–49.5)	0.66 (0.36–1.21)
HR status				
ER- and/or PgR-positive	72	41 (56.9)	18.9 (14.7–33.1)	–
ER- and PgR-negative	60	29 (48.3)	33.7 (20.6–NE)	0.79 (0.49–1.27)
Menopausal status				
Premenopausal	46	27 (58.7)	22.8 (16.6–49.5)	–
Postmenopausal	86	43 (50.0)	22.8 (14.6–NE)	1.05 (0.65–1.70)
Recurrence				
De novo metastatic disease	83	48 (57.8)	20.7 (16.6–37.5)	–
Recurrent metastatic disease^b^	49	22 (44.9)	28.1 (16.7–NE)	0.77 (0.47–1.28)

*CI* confidence interval, *ER* estrogen receptor, *HER2* human epidermal growth factor receptor 2, *HR* hazard ratio, *IHC* immunohistochemistry, *ISH* in situ hybridization, *PFS* progression-free survival, *PgR* progesterone receptor

^a^Includes prior trastuzumab and/or chemotherapy (patients receiving hormonal therapy alone are included in the ‘No’ population)

^b^Patients with non-recurrence were classified as having "de novo metastatic disease”

### Treatment exposure

Median duration of study treatment was 17.1 months (range 1.3–60.9) at final analysis. A total of 104 patients (78.8%) completed six cycles of docetaxel treatment; 48 patients (46.2%) discontinued docetaxel at Cycle 6 and 56 patients (53.8%) continued docetaxel beyond Cycle 6. Reasons for permanent discontinuation of docetaxel are presented in Online Resource 10.

By final analysis, 75 patients (56.8%) had withdrawn from study treatment due to lack of efficacy. Other reasons for withdrawal included withdrawal by patient (*n* = 12 [9.1%]), adverse events (*n* = 9 [6.8%]), and other reasons (patients who discontinued due to reasons unrelated to investigational medicinal product efficacy or safety; *n* = 36 [27.3%]).

### Safety

**Fig. 3:**
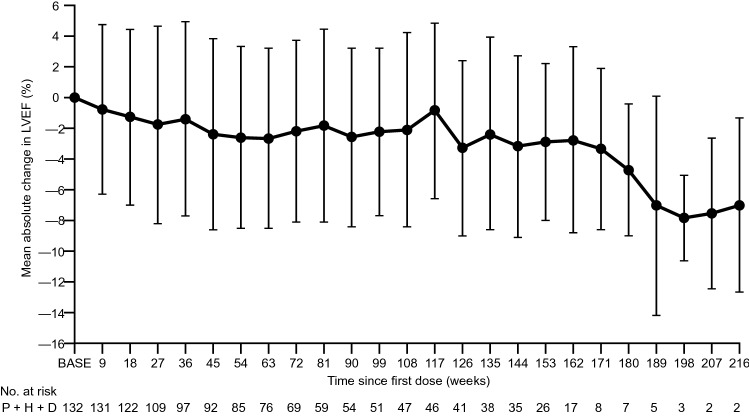
Time-dependent change in LVEF

Adverse events of any grade occurred in all patients, with a total of 3789 events reported. The most common any-grade adverse events occurring in ≥ 30% of patients were alopecia (*n* = 122 [92.4%]), diarrhea (*n* = 104 [78.8%]), and decreased neutrophil count (*n* = 83 [62.9%]) (Table 4). Grade ≥ 3 adverse events occurred in 127 patients (96.2%). The most common grade ≥ 3 adverse events occurring in ≥ 5% of patients were decreased neutrophil count (*n* = 82 [62.1%]), decreased white blood cell count (*n* = 67 [50.8%]), and febrile neutropenia (*n* = 42 [31.8%]) (Table 4). Any-grade and grade ≥ 3 peripheral neuropathy occurred in 50 patients (37.9%) and 1 patient (0.8%), respectively. Serious adverse events occurred in 33 patients (25.0%), with febrile neutropenia (*n* = 8; 6.1%) as the only serious adverse event that occurred in ≥ 5% of patients. Treatment-related adverse events occurred in 132 patients (100%). Adverse events leading to the discontinuation of the study drug were reported in 62 patients (47.0%) and adverse events leading to dose modification or interruption in 98 patients (74.2%). With regard to cardiac safety, only seven patients (5.3%) experienced a significant decline in LVEF (LVEF decrease ≥ 10% from baseline to < 50%) (Fig. 3). There were no adverse event-related deaths.

**Table 4:** Summary of adverse events, including all where any grade occurred in ≥ 30% or grade ≥ 3 occurred in ≥ 5%

Adverse event	Patients, *n* (%) (*N* = 132)	
	Any grade	Grade ≥ 3
Alopecia	122 (92.4)	0
Diarrhea	104 (78.8)	6 (4.5)
Neutrophil count decreased	83 (62.9)	82 (62.1)
Stomatitis	81 (61.4)	4 (3.0)
Taste abnormality	75 (56.8)	0
White blood cell count decreased	70 (53.0)	67 (50.8)
Loss of appetite	70 (53.0)	7 (5.3)
Nasopharyngitis	69 (52.3)	0
Malaise	65 (49.2)	0
Rash	63 (47.7)	1 (0.8)
Nausea	60 (45.5)	1 (0.8)
Peripheral edema	57 (43.2)	1 (0.8)
Injection-site reaction	53 (40.2)	0
Peripheral neuropathy	50 (37.9)	1 (0.8)
Muscle pain	50 (37.9)	1 (0.8)
Fever	45 (34.1)	1 (0.8)
Vomiting	42 (31.8)	1 (0.8)
Febrile neutropenia	42 (31.8)	42 (31.8)
Neutropenia	32 (24.2)	32 (24.2)
Anemia	22 (16.7)	9 (6.8)
Leukopenia	14 (10.6)	14 (10.6)

## Discussion

In this phase IV, multicenter, single-arm study of pertuzumab plus trastuzumab and docetaxel, the investigator-assessed PFS, OS, and investigator-assessed ORR and disease control rate results suggest that the treatment combination is effective in Japanese patients, similar to the overall population assigned to the pertuzumab-treated arm in the pivotal CLEOPATRA study [9, 11].

Investigator-assessed PFS in the Japanese patients included in our study was numerically longer than the IRC-assessed PFS results observed in the overall population in the CLEOPATRA study (median PFS: 22.8 months vs. 18.5 months) [9, 11]. The slightly longer PFS observed in COMACHI compared with CLEOPATRA may be due to differences in patient characteristics between the two studies [9]. For example, COMACHI included a higher proportion of patients with non-visceral disease (although studies have reported comparable response rates for patients with or without visceral disease when treated with various chemotherapeutic agents) [13], a lower proportion of patients who received prior treatment with anthracyclines, and a lower proportion of patients with an ECOG performance status of 1. Similar to PFS, the results for time to response and DoR in our study also suggest that pertuzumab plus trastuzumab and docetaxel is effective in Japanese patients; the median DoR observed here was greater than that observed in the overall population of the CLEOPATRA 3-year follow-up study (26.3 months [95% CI 17.1–NE] vs. 20.2 months [95% CI 16.0–24.0]) [14].

As expected, subgroup analysis demonstrated a lower median PFS in patients with visceral metastasis, compared to those with non-visceral disease. Interestingly, the analysis also found a lower median PFS in patients with a positive hormone-receptor status, compared to those with a negative status. Overall, this suggests that it may be meaningful to consider adding hormone therapy to patients of this subgroup. In support of this, the recent PERTAIN study examining the efficacy of pertuzumab plus trastuzumab and an aromatase inhibitor (anastrozole or letrozole) in patients with HER2-positive and hormone-receptor-positive, metastatic/locally advanced breast cancer met its primary PFS endpoint (median PFS: 18.9 months vs. 15.8 months for trastuzumab alone; stratified HR 0.65; 95% CI 0.48–0.89; *P* < 0.01) [15]. Improved therapeutic outcomes of combined hormonal and trastuzumab therapy in this patient population have also been demonstrated in randomized phase III clinical trials, for example, the TAnDEM and eLEcTRA trials [16, 17]. Interestingly, discontinuation of docetaxel at Cycle 6 in our study did not reduce PFS in patients compared to those who continued beyond Cycle 6, suggesting that withdrawing docetaxel at Cycle 6 may reduce toxicity in patients without worsening prognosis. However, these analyses should be interpreted with caution because of their exploratory nature and non-randomized comparison with potential selection bias. Moreover, in contrast to our study, a systematic review of randomized controlled trials of first-line chemotherapy in patients with metastatic breast cancer found longer chemotherapy durations (≥ 6 cycles) to result in clinically meaningful improvements in PFS [18]. This discussion is particularly relevant given that there are currently no specific recommendations in the literature regarding how long chemotherapy should be continued after treatment response and while toxicity is manageable.

Study treatment exposure was similar between COMACHI and CLEOPATRA, with median durations of study treatment of 17.1 months and 18.1 months, respectively [9]. Treatment with pertuzumab plus trastuzumab and docetaxel was generally well tolerated in this study, with no new safety signals detected. There were also no clinically problematic changes in laboratory parameters, cardiac function, or vital signs in our study. Of note, incidence of febrile neutropenia and diarrhea occurred more frequently in COMACHI than in the pertuzumab arm of the primary CLEOPATRA study (31.8% vs. 13.8% and 78.8% vs. 66.8%, respectively) [9]. However, febrile neutropenia resulted in discontinuation of the study and concomitant medication in only one patient in COMACHI. Furthermore, the prophylactic use of granulocyte-colony stimulating factor in Japanese patients was approved in 2014; therefore, the higher incidence of febrile neutropenia in this study is not thought to be a significant concern in practice. In support of this, in the phase III randomized PEONY trial of Asian patients with early/locally advanced HER2 breast cancer treated with pertuzumab plus trastuzumab and docetaxel, concomitant use of prophylactic granulocyte-colony stimulating factor was permitted, with only four patients (1.8%) reported to develop febrile neutropenia [19]. Similar to the results observed in our study, a subgroup analysis of Asian patients in CLEOPATRA demonstrated a higher incidence of febrile neutropenia in Asian patients than in patients from other regions [20]. In contrast, Chinese patients with locally recurrent or metastatic HER2-positive breast cancer in the PUFFIN study appeared to have a lower incidence of febrile neutropenia than reported here and in the Asian and overall population in the CLEOPATRA study [21].

As this was a single-arm study, no comparator arm is available to allow for direct comparison of pertuzumab plus trastuzumab and docetaxel with placebo plus trastuzumab and docetaxel. Furthermore, although the similarities in study design and enrolled patient populations between COMACHI and CLEOPATRA have allowed some comparisons to be made, these should be interpreted with caution in light of their retrospective nature. Moreover, the present study did not include assessments of health-related quality of life, which has been shown to be crucial to overall patient outcomes. For example, a study of female survivors of breast cancer found that women with the highest social well-being quality-of-life score had a significantly reduced risk of mortality (95% CI 0.46–0.85; *P* = 0.002) and disease recurrence (95% CI 0.38–0.71; *P* < 0.001) [22]. However, in light of the efficacy and safety profile observed in COMACHI, it is reasonable to expect positive quality-of-life scores in Japanese patients receiving pertuzumab plus trastuzumab and docetaxel, as reported in the overall population in CLEOPATRA [23]. Finally, all patients in COMACHI received pertuzumab plus trastuzumab and docetaxel regardless of treatment-free intervals; patients with a short treatment-free interval whose disease has progressed on trastuzumab may be given alternative therapy options such as ado-trastuzumab emtansine [24]. However, it should be noted that ado-trastuzumab emtansine had not yet been approved in Japan upon initiation of the COMACHI study.

In conclusion, the results from the COMACHI study suggest a similar efficacy and safety profile for pertuzumab plus trastuzumab and docetaxel in Japanese patients compared with patients in the overall population of the CLEOPATRA study. These results provide further evidence to support pertuzumab plus trastuzumab and docetaxel combination therapy as the standard of care for first-line treatment of Japanese patients with inoperable or recurrent HER2-positive breast cancer.

## Electronic supplementary material

Below is the link to the electronic supplementary material.

Supplementary file1 (PDF 947 kb)

Supplementary file2 (PDF 98 kb)

## Data Availability

Qualified researchers may request access to individual patient level data through the clinical study data request platform (www.clinicalstudydatarequest.com). For further details on Chugai’s Data Sharing Policy and how to request access to related clinical study documents, see here (www.chugai-pharm.co.jp/english/profile/rd/ctds_request.html).
