# Supplementary material for: The efficacy and safety of pertuzumab plus trastuzumab and docetaxel as a first-line therapy in Japanese patients with inoperable or recurrent HER2-positive breast cancer: the COMACHI study
Source: Breast Cancer Res Treat. 2020 Sep 13;185(1):125–34. doi: 10.1007/s10549-020-05921-x (PMC7843485; doi:10.1007/s10549-020-05921-x)
Supplement: Supplementary file 1 — Supplementary file1 (PDF 947 kb) [file 10549_2020_5921_MOESM1_ESM.pdf]

Electronic supplementary material

Title: The efficacy and safety of pertuzumab plus trastuzumab and docetaxel as a first-line therapy in Japanese patients with inoperable or recurrent HER2-positive breast cancer: the COMACHI study

Target journal: *Breast Cancer Research and Treatment*

Authors: Masato Takahashi[1] • Shoichiro Ohtani[2] • Shigenori E. Nagai[3] • Seiki Takashima[4] • Miki Yamaguchi[5] • Michiko Tsuneizumi[6] • Yoshifumi Komoike[7] • Tomofumi Osako[8] • Yoshinori Ito[9] • Masahiko Ikeda[10] • Kazushige Ishida[11] • Takahiro Nakayama[12] • Tsutomu Takashima[13] • Takashi Asakawa[14] • Sho Matsumoto[15] • Daisuke Shimizu[16] • Norikazu Masuda[17]

Author affiliations:

[1] Department of Breast Surgery, National Hospital Organization Hokkaido Cancer Center, Sapporo, Japan (masato.takahashi0725@gmail.com)

[2] Breast surgery, Hiroshima City Hiroshima Citizens Hospital, Hiroshima, Japan (sho_ohtani@hotmail.com)

[3] Breast Oncology, Saitama Cancer Center, Saitama, Japan (snagai@cancer-c.pref.saitama.jp)

[4] Breast Oncology, National Hospital Organization Shikoku Cancer Center, Matsuyama, Japan (takashima.seiki.ch@mail.hosp.go.jp)

[5] Breast surgery, JCHO Kurume General Hospital, 21 Kushihara-machi Kurume Fukuoka, Japan (yamaguchi-miki@kurume.jcho.go.jp)

[6] Breast surgery, Shizuoka General Hospital, Shizuoka, Japan (m-tsuneizumi@i.shizuoka-pho.jp)

[7] Surgery, Kindai University Hospital, Osakasayama, Japan (komoike@med.kindai.ac.jp)

[8] Breast Center, Kumamoto Shinto General Hospital, Kumamoto, Japan (osako@k-shinto.or.jp)

[9] Breast Medical Oncology, Cancer Institute Hospital of JFCR, Tokyo, Japan (yito@jfcr.or.jp)

[10] Breast and Thyroid Surgery, Fukuyama City Hospital, Hiroshima, Japan (masahikoikeda@city.fukuyama.hiroshima.jp)

[11] Surgery, Iwate Medical University, 2-1-1, Idaidori, Yahaba-cho, Shiwa-gun, Iwate Prefecture 028-3695, Japan (kishiday08@gmail.com)

[12] Breast and Endocrine Surgery, Osaka International Cancer Institute, 3-1-69 Otemae, Chuo-ku, Osaka 541-8567, Japan (nakayama-ta@mc.pref.osaka.jp)

[13] Breast and Endocrine Surgery, Osaka City University Graduate School of Medicine, 1-4-3 Asahimachi Abeno-ku Osaka 5458585, Japan (tsutomu-@rd5.so-net.ne.jp)

[14] Clinical Information and Intelligence Department, Chugai Pharmaceutical Co., Ltd, 2-chome-1-1, Nihonbashi-Muromachi, Chuo City, Tokyo 103-8324, Japan (asakawatks@chugai-pharm.co.jp)

[15] Clinical Study Management Department, Chugai Pharmaceutical Co., Ltd, 2-chome-1-1, Nihonbashi-Muromachi, Chuo City, Tokyo 103-8324, Japan (matsumoto.sho90@chugai-pharm.co.jp)

[16] Clinical Science and Strategy Department, Chugai Pharmaceutical Co., Ltd, 1-1 Nihonbashi-Muromachi 2-Chome, Chuo-ku, Tokyo 103-8324, Japan (shimizu.daisuke29@chugai-pharm.co.jp)

[17] Surgery, Breast Oncology, National Hospital Organization Osaka National Hospital, Osaka, Japan (nmasuda@alpha.ocn.ne.jp)

Corresponding author: Norikazu Masuda, Surgery, Breast Oncology, National Hospital Organization Osaka National Hospital, Osaka, Japan, Telephone: +81-669421331; Fax: +81-669463608; Email address: nmasuda@alpha.ocn.ne.jp; ORCID ID: https://orcid.org/0000-0002-7302-0278

Online Resource 1 Final analysis of PFS by hormone receptor status (clinical cutoff date: April 25, 2019)

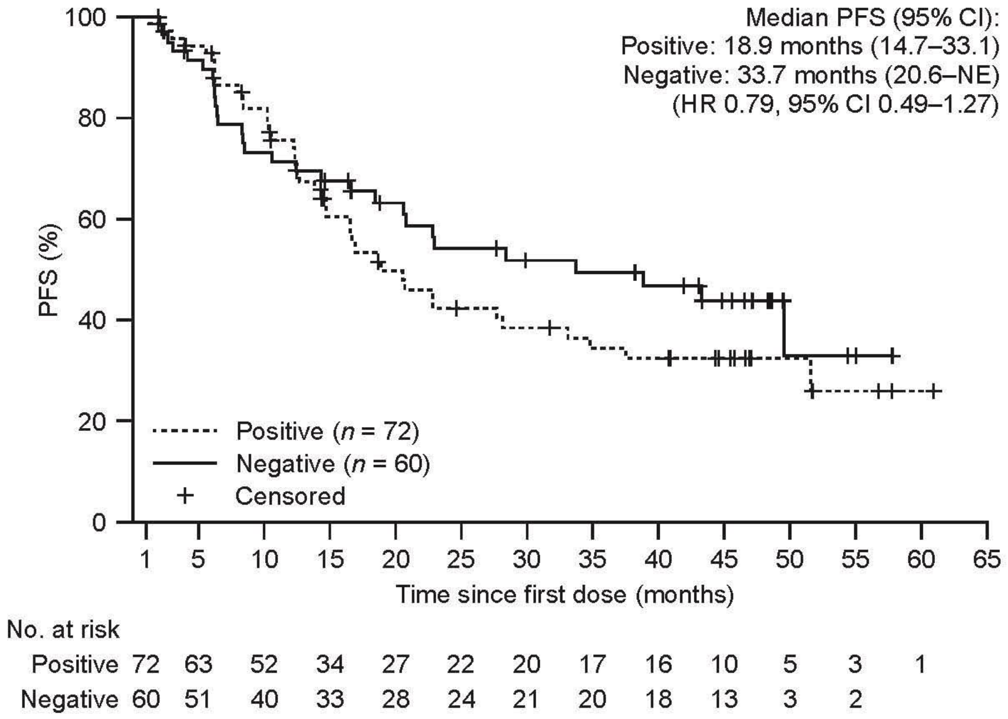

CI confidence interval, *HR* hazard ratio, *NE* not evaluable, *PFS* progression-free survival

Online Resource 2 Final analysis of PFS in patients with visceral vs. non-visceral disease (clinical cutoff date: April 25, 2019)

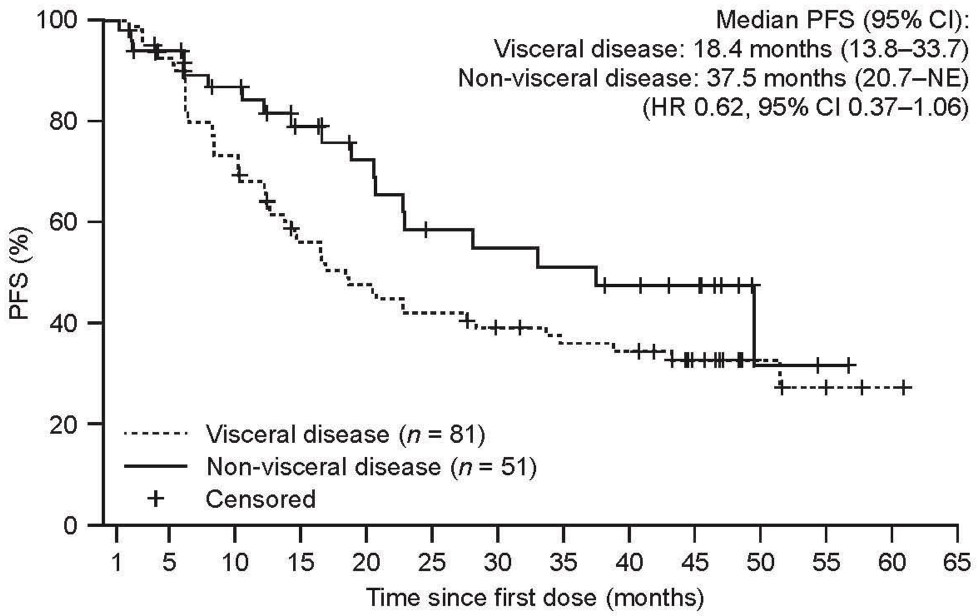

No. at risk													
Visceral disease	81	73	57	40	34	30	26	23	22	13	6	4	1
Non-visceral disease	51	41	35	27	21	16	15	14	12	10	2	1	

CI confidence interval, *HR* hazard ratio, *NE* not evaluable, *PFS* progression-free survival

Online Resource 3 Final analysis of PFS by age (< 65 or ≥ 65 years old) (clinical cutoff date: April 25, 2019)

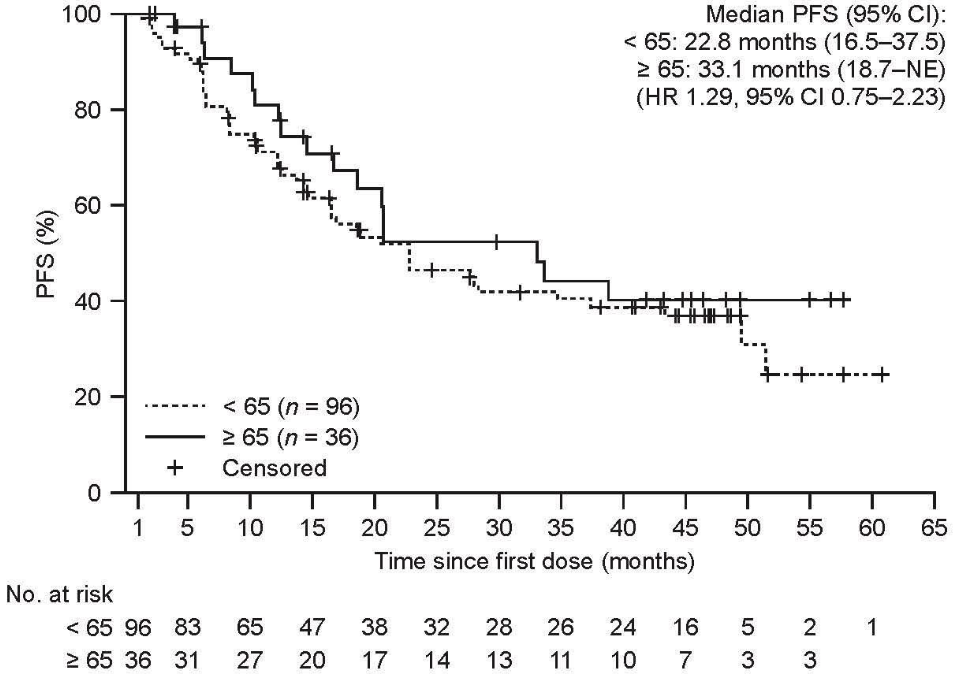

CI confidence interval, *HR* hazard ratio, *NE* not evaluable, *PFS* progression-free survival

Online Resource 4 Final analysis of PFS by HER2 status (clinical cutoff date: April 25, 2019)

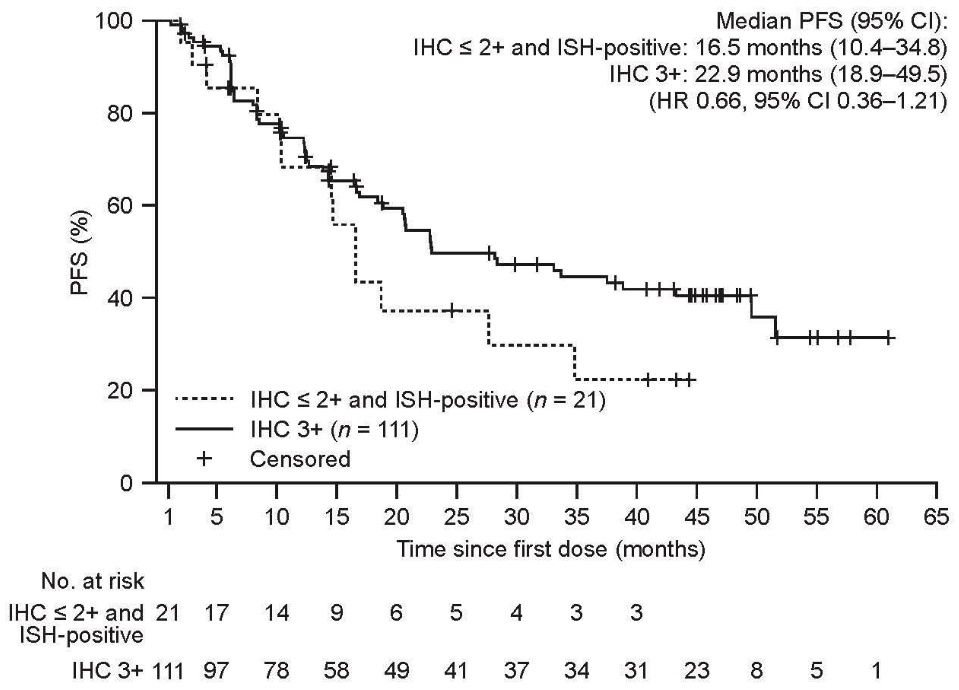

CI confidence interval, HER2 human epidermal growth factor receptor 1, *HR* hazard ratio, *IHC* immunohistochemistry, *ISH* in situ hybridization, *NE* not evaluable, *PFS* progression-free survival

Online Resource 5 Final analysis of PFS by prior neo(adjuvant) therapy (clinical cutoff date: April 25, 2019)

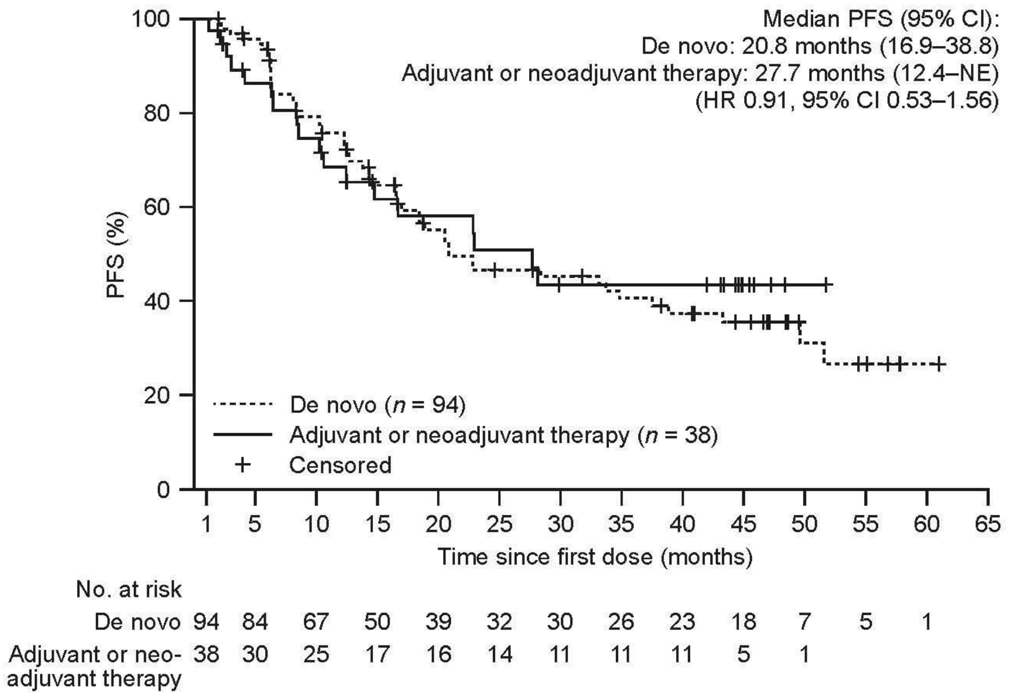

CI confidence interval, *HR* hazard ratio, *NE* not evaluable, *PFS* progression-free survival

Online Resource 6 Final analysis of PFS by recurrence (clinical cutoff date: April 25, 2019)

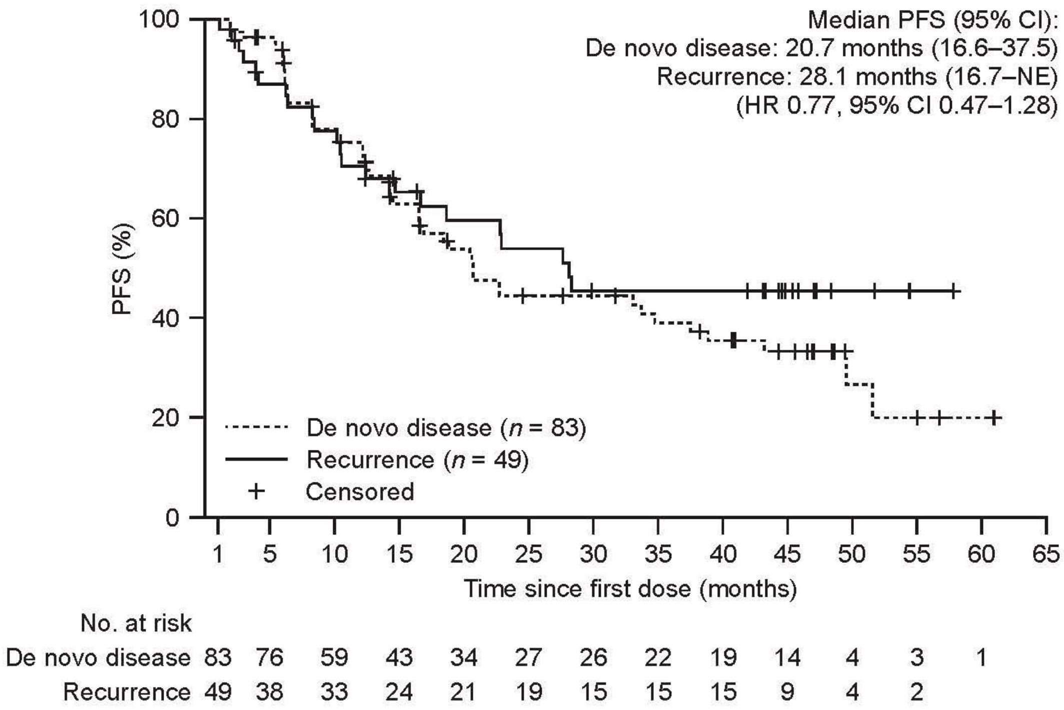

CI confidence interval, *HR* hazard ratio, *NE* not evaluable, *PFS* progression-free survival

Online Resource 7 Final analysis of PFS by menopausal status (clinical cutoff date: April 25, 2019)

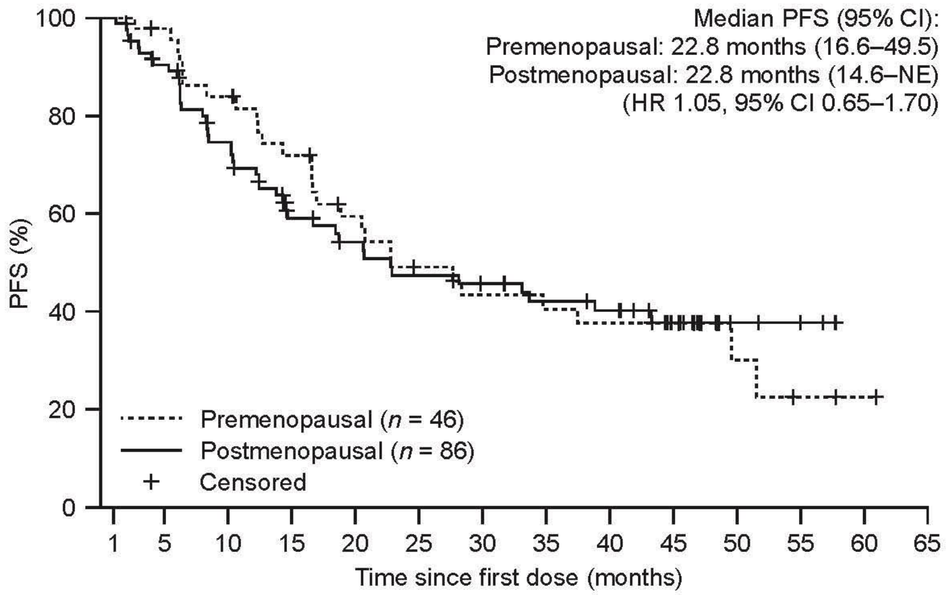

No. at risk													
Premenopausal	46	42	36	30	23	18	15	14	13	12	4	2	1
Postmenopausal	86	72	56	37	32	28	26	23	21	11	4	3	

CI confidence interval, *HR* hazard ratio, *NE* not evaluable, *PFS* progression-free survival

Online Resource 8 Final analysis of PFS by docetaxel discontinuation at or beyond Cycle 6 (clinical cutoff date: April 25, 2019)

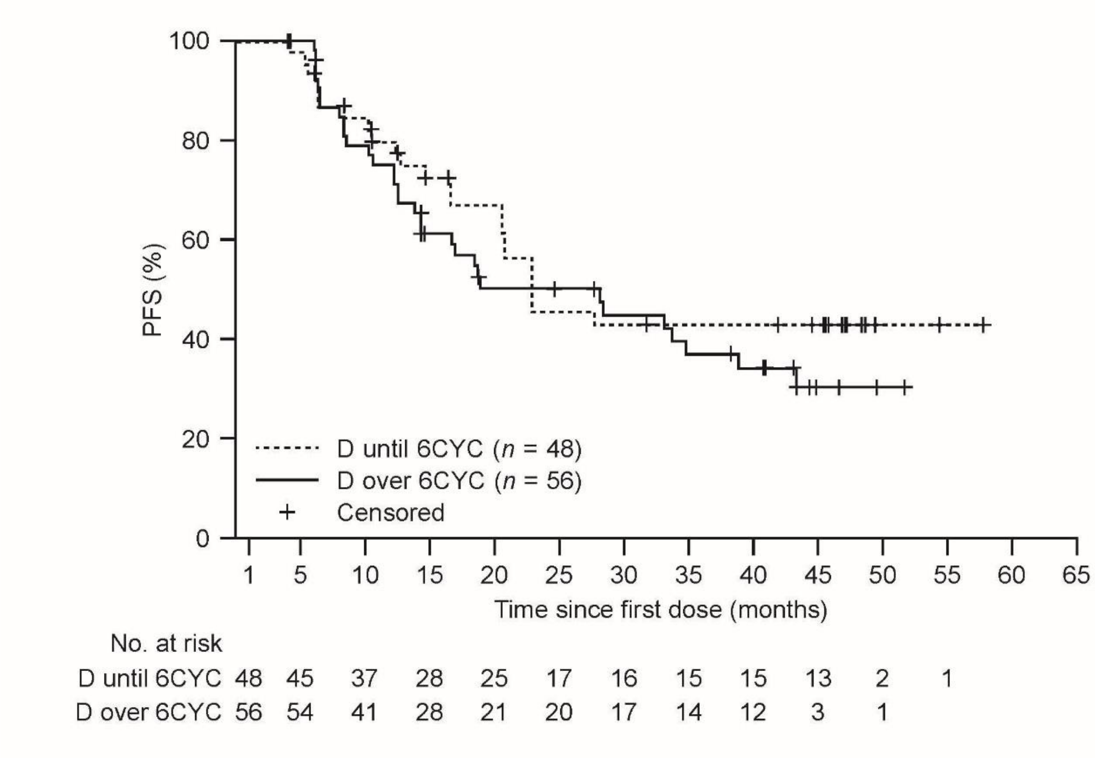

6CYC Cycle 6, *D* docetaxel, *PFS* progression-free survival

Online Resource 9 Final analysis of PFS by docetaxel discontinuation at or beyond Cycle 6, excluding discontinuation due to adverse event or lack of efficacy (clinical cutoff date: April 25, 2019)

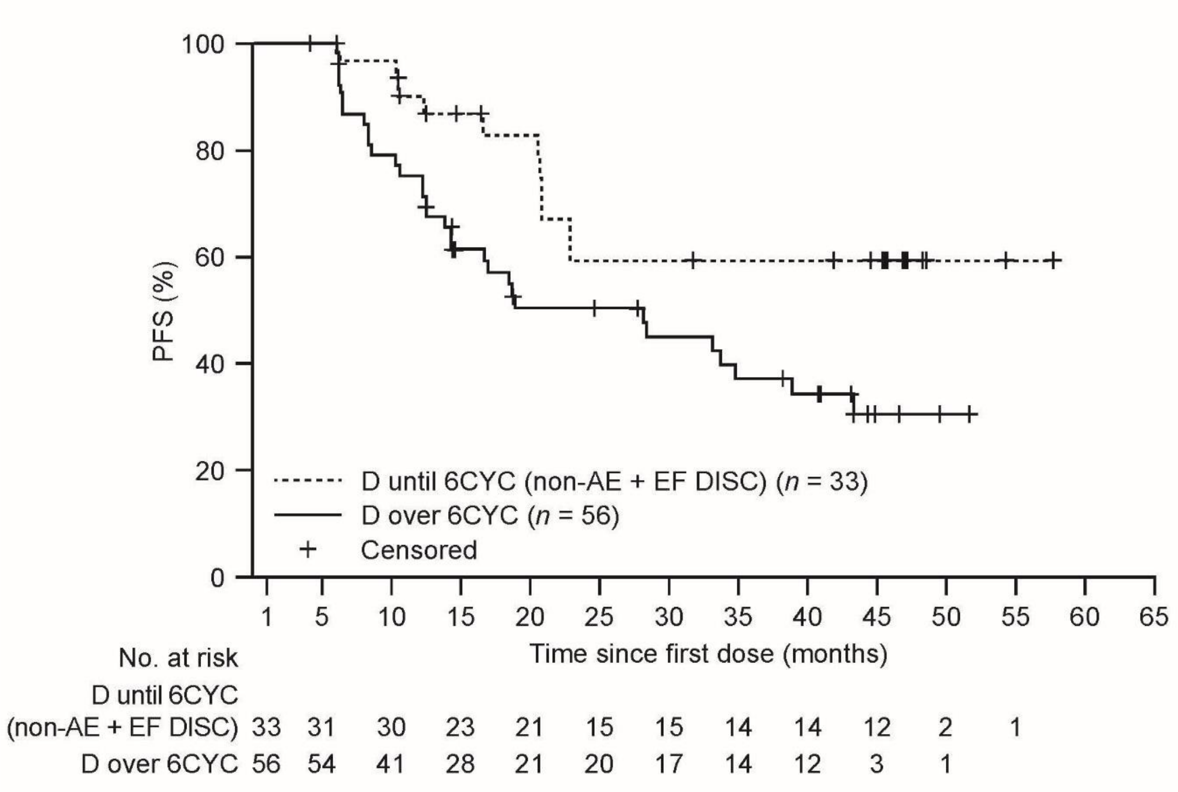

AE adverse event, *6CYC* Cycle 6, *D* docetaxel, *DISC* discontinuation, *EF* efficacy, *PFS* progression-free survival
