# Supplementary material for: The efficacy and safety of pertuzumab plus trastuzumab and docetaxel as a first-line therapy in Japanese patients with inoperable or recurrent HER2-positive breast cancer: the COMACHI study
Source: Breast Cancer Res Treat. 2020 Sep 13;185(1):125–34. doi: 10.1007/s10549-020-05921-x (PMC7843485; doi:10.1007/s10549-020-05921-x)
Supplement: Supplementary file 2 — Supplementary file2 (PDF 98 kb) [file 10549_2020_5921_MOESM2_ESM.pdf]

Electronic supplementary material

Title: The efficacy and safety of pertuzumab plus trastuzumab and docetaxel as a first-line therapy in Japanese patients with inoperable or recurrent HER2-positive breast cancer: the COMACHI study

Target journal: *Breast Cancer Research and Treatment*

Authors: Masato Takahashi[1] • Shoichiro Ohtani[2] • Shigenori E. Nagai[3] • Seiki Takashima[4] • Miki Yamaguchi[5] • Michiko Tsuneizumi[6] • Yoshifumi Komoike[7] • Tomofumi Osako[8] • Yoshinori Ito[9] • Masahiko Ikeda[10] • Kazushige Ishida[11] • Takahiro Nakayama[12] • Tsutomu Takashima[13] • Takashi Asakawa[14] • Sho Matsumoto[15] • Daisuke Shimizu[16] • Norikazu Masuda[17]

Author affiliations:

[1] Department of Breast Surgery, National Hospital Organization Hokkaido Cancer Center, Sapporo, Japan (masato.takahashi0725@gmail.com)

[2] Breast surgery, Hiroshima City Hiroshima Citizens Hospital, Hiroshima, Japan (sho_ohtani@hotmail.com)

[3] Breast Oncology, Saitama Cancer Center, Saitama, Japan (snagai@cancer-c.pref.saitama.jp)

[4] Breast Oncology, National Hospital Organization Shikoku Cancer Center, Matsuyama, Japan (takashima.seiki.ch@mail.hosp.go.jp)

[5] Breast surgery, JCHO Kurume General Hospital, 21 Kushihara-machi Kurume Fukuoka, Japan (yamaguchi-miki@kurume.jcho.go.jp)

[6] Breast surgery, Shizuoka General Hospital, Shizuoka, Japan (m-tsuneizumi@i.shizuoka-pho.jp)

[7] Surgery, Kindai University Hospital, Osakasayama, Japan (komoike@med.kindai.ac.jp)

[8] Breast Center, Kumamoto Shinto General Hospital, Kumamoto, Japan (osako@k-shinto.or.jp)

[9] Breast Medical Oncology, Cancer Institute Hospital of JFCR, Tokyo, Japan (yito@jfcr.or.jp)

[10] Breast and Thyroid Surgery, Fukuyama City Hospital, Hiroshima, Japan (masahikoikeda@city.fukuyama.hiroshima.jp)

[11] Surgery, Iwate Medical University, 2-1-1, Idaidori, Yahaba-cho, Shiwa-gun, Iwate Prefecture 028-3695, Japan (kishiday08@gmail.com)

[12] Breast and Endocrine Surgery, Osaka International Cancer Institute, 3-1-69 Otemae, Chuo-ku, Osaka 541-8567, Japan (nakayama-ta@mc.pref.osaka.jp)

[13] Breast and Endocrine Surgery, Osaka City University Graduate School of Medicine, 1-4-3 Asahimachi Abeno-ku Osaka 5458585, Japan (tsutomu-@rd5.so-net.ne.jp)

[14] Clinical Information and Intelligence Department, Chugai Pharmaceutical Co., Ltd, 2-chome-1-1, Nihonbashi-Muromachi, Chuo City, Tokyo 103-8324, Japan (asakawatks@chugai-pharm.co.jp)

[15] Clinical Study Management Department, Chugai Pharmaceutical Co., Ltd, 2-chome-1-1, Nihonbashi-Muromachi, Chuo City, Tokyo 103-8324, Japan (matsumoto.sho90@chugai-pharm.co.jp)

[16] Clinical Science and Strategy Department, Chugai Pharmaceutical Co., Ltd, 1-1 Nihonbashi-Muromachi 2-Chome, Chuo-ku, Tokyo 103-8324, Japan (shimizu.daisuke29@chugai-pharm.co.jp)

[17] Surgery, Breast Oncology, National Hospital Organization Osaka National Hospital, Osaka, Japan (nmasuda@alpha.ocn.ne.jp)

Corresponding author: Norikazu Masuda, Surgery, Breast Oncology, National Hospital Organization Osaka National Hospital, Osaka, Japan, Telephone: +81-669421331; Fax: +81-669463608; Email address: nmasuda@alpha.ocn.ne.jp; ORCID ID: https://orcid.org/0000-0002-7302-0278

Online Resource 10 Reasons for discontinuation of docetaxel in patients

Reason for discontinuation of D	D duration, *n* (%)		
	Before Cycle 6	At Cycle 6	After Cycle 6
	n = 28	*n* = 48	*n* = 56
Adverse event	19 (67.9)	14 (29.2)	28 (50.0)
Lack of efficacy	6 (21.4)	1 (2.1)	11 (19.6)
Withdrawal of consent by patient	1 (3.6)	10 (20.8)[a]	3 (5.4)
Doctor's decision	1 (3.6)	24 (50.0)[a]	10 (17.9)
Other	1 (3.6)	0	4 (7.1)

D docetaxel

[a]One patient discontinued for both of these reasons.
